# 

**Published:** 1892-07

**Authors:** 


### CONTENTS.

ORIGINAL ARTICLES—Appendicitis, Typhitis and Perityphlitic Abscess —By A. H. Levings, M. D., Milwaukee........................367
Remarks on the Nature and Treatment of Tuberculosis—By E. L. Shurly, M. D., Detroit, Mich........................382
Twin Pregnancy—One Fœtus in Utero and the other Extra Uterine—By E. W. Martin, M. D., Frement, Neb........................392
CORRESPONDENCE—Our New York Letter........................396
A Growing Evil which should be Remedied........................399
SOCIETY NOTES—Clinical Society of Maryland........................401
American Medical Association........................405
BOOK REVIEWS........................408
A Treatise on Practical Anatomy—By Henry C. Boenning, M. D.
Synopsis of Human Anatomy—By James K. Young, M. D.
Essential of Minor Surgery and Bandaging—By Edward Martin, M. D.
SELECTIONS AND ABSTRACTS—Acute Angina following the Use of Salol.381
Burned Flour as a Day Dressing for Ill-Conditioned Wounds, Erysipelas, etc........................391
Rapid Dilatation of the Uterus, for Hemorrhage........................409
Epididymitis........................409
Application of Forceps........................410
The Disinfection and Sterilization of Surgical Instruments........................411
Treatment of Dysentery........................412
Does Ether assist Digestion?
EDITORIAL........................414–418
American Medical Association—A Reply—Death of Dr. Kennedy—Laying of Corner-Stone, Southern Medical College—Charlotte Medical Journal—Dr. Whelpley—Dr. Geo. T. Brown.
PRESCRIPTION DEPARTMANT........................421–422
Pruritis Ani et Vulvæ—Diarrhœa of Indigestion, etc.—In Typhoid Fever—Irritated Dyspepsia with Constipation—Spasmodic Asthma—Typhoid Fever—Impotence—Cholera Infantum—Treatment of Baldness—Infantile Diarrhœa.
SPECIAL NOTES........................419–42
0

